# MT4-MMP: The GPI-Anchored Membrane-Type Matrix Metalloprotease with Multiple Functions in Diseases

**DOI:** 10.3390/ijms20020354

**Published:** 2019-01-16

**Authors:** Cassandre Yip, Pierre Foidart, Agnès Noël, Nor Eddine Sounni

**Affiliations:** Laboratory of Tumor and Development Biology, Groupe Interdisciplinaire de Génoprotéomique Appliqué-Cancer (GIGA-Cancer), University of Liège, Liège 4000, Belgium; cassandre.yip@uliege.be (C.Y.); pierre.foidart@chuliege.be (P.F.); agnes.noel@uliege.be (A.N.)

**Keywords:** MT4-MMP, cancer, diseases

## Abstract

MT4-MMP (or MMP17) belongs to the Membrane-Type Matrix Metalloproteinase (MT-MMP) family. This family of proteases contributes to extracellular matrix remodeling during several physiological processes, including embryogenesis, organogenesis, tissue regeneration, angiogenesis, wound healing, and inflammation. MT4-MMP (MMP17) presents unique characteristics compared to other members of the family in terms of sequence homology, substrate specificity, and internalization mode, suggesting distinct physiological and pathological functions. While the physiological functions of MT4-MMP are poorly understood, it has been involved in different pathological processes such as arthritis, cardiovascular disease, and cancer progression. The *mt4-mmp* transcript has been detected in a large diversity of cancers. The contribution of MT4-MMP to tumor development has been further investigated in gastric cancer, colon cancer, head and neck cancer, and more deeply in breast cancer. Given its contribution to different pathologies, particularly cancers, MT4-MMP represents an interesting therapeutic target. In this review, we examine its biological and structural properties, and we propose an overview of its physiological and pathological functions.

## 1. Introduction

The integrity of interstitial compartments is crucial for tissue homeostasis. The perturbation of the extracellular matrix and its related components destabilizes this balance, leading to pathogenesis. Matrix Metalloproteinases (MMPs) are the main remodeling enzymes of the extracellular matrix. This protease family counts more than 20 members, and most of them are secreted in the extracellular microenvironment. The membrane-type MMP (MT-MMPs) are associated to the membrane by a transmembrane domain, an amino-terminal link, or a glycosylphosphatidylinositol anchor (GPI). Together, secreted or attached to the membrane, MMPs can directly cleave almost all components of the extracellular matrix (ECM). However, the GPI anchor confers to MMPs a unique location in the lipid raft, giving access to a specific set of substrates. Only two MT-MMPs display this anchor: MT4-MMP (MMP-17) and MT6-MMP (MMP-25). In this review, we focus on MT4-MMP. Discovered more than 20 years ago, this protease aroused interest only a decade ago [1,2,3,4]. MT4-MMP exhibits unique characteristics, which distinguishes it from other MMPs. Unlike the others, it is unable to activate pro-MMP2 and cleaves only a few ECM components [5,6]. Its sensitivity to tissue inhibitors of metalloproteinases (TIMPs) is also different, with MT4-MMP being more sensitive to TIMP1 than TIMP2 [6,7]. These differences are probably due to the least degree of sequence identity [1]. MT4-MMP has been involved in inflammation and angiogenesis, contributing to associated pathologies such as osteoarthritis and atherosclerosis, as well as thoracic aortic aneurysms and dissection (TAAD) [2,8,9]. Interestingly, inflammation and angiogenesis are two pillars of tumor development. The *mt4-mmp* transcript has been detected in prostate carcinomas, oral carcinomas, osteosarcomas, gastrointestinal adenocarcinomas, embryonal carcinomas, leukemias, lung carcinomas, glioblastomas, cervical carcinomas, melanomas, adrenal adenocarcinomas, and thyroid carcinomas [3,10,11,12]. MT4-MMP was first described in breast cancers [1], in which it has been more widely investigated compared to the other cancers. The pro-angiogenic and pro-metastatic functions of MT4-MMP have been highlighted in breast cancer [4,13]. MT4-MMP-mediated metastatic dissemination has been also pointed out in colon cancer and head and neck cancer [3,11]. All these data propel MT4-MMP onto the stage of future potential therapeutic treatments.

## 2. Characteristics of the MMP family

**Figure 1:**
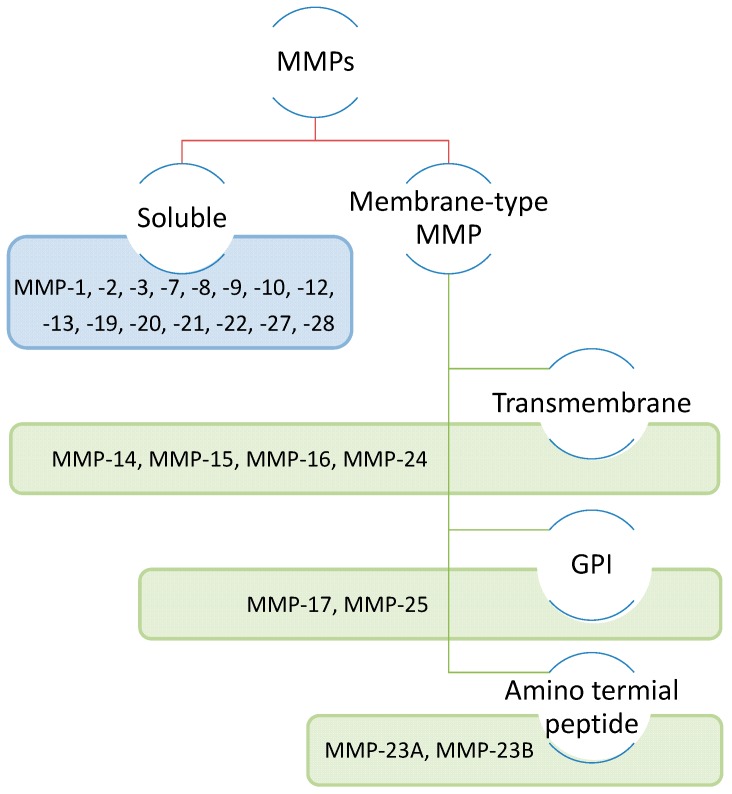
Classification of different Matrix Metalloproteinases (MMPs) according to their structure. Matrix Metalloproteases are either soluble (MMPs) or membrane-tethered (MT-MMPs). MMP14 (MT1-MMP), MMP15 (MT2-MMP), MMP16 (MT3-MMP), and MMP24 (MT5-MMP) are attached to the cell membrane by a transmembrane domain. MMP17 (MT4-MMP) and MMP25 (MT6-MMP) are linked to the cell membrane by a glycosylphosphatidylinositol anchor (GPI).

The MMPs are endopeptidases characterized by the presence of a zinc ion in the catalytic domain. Twenty-four members have been identified and are separated into two different groups: The soluble MMPs (MMP-1, -2, -3, -7, -8, -9, -10, -11, -12, -13, -19, -20, -21, -22, -27, and -28) and the MMPs linked to the membrane by a transmembrane domain (MMP-14, -15, -16, and -24), a glycosylphosphatidylinositol (GPI) anchor (MMP-17 and -25), or an amino-terminal signal peptide (MMP-23A and -23B). The groups are shown in Figure 1.

**Figure 2:**
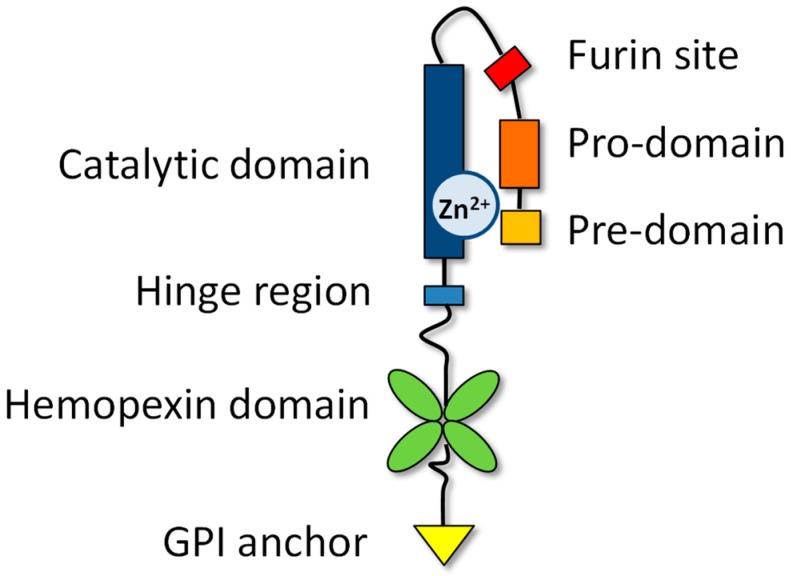
Structural domains of MT4-MMP, including the pre-domain or signal peptide (amino acids 1 to 41), the pro-domain (42–128), the catalytic domain with a zinc ion (129–297), a linker (298–333) containing the furin site (R–X–K/R–R), the hemopexin domain (334–535), and the glycosylphosphatidylinositol (GPI) anchored to the membrane (572–605).

The MMPs share common structures including: (1) The pre-domain, an N-terminal sequence driving the MMP to the endoplasmic reticulum (ER); (2) the pro-domain, keeping enzymes in an inactive form; and (3) the catalytic domain, implicated in the recognition and cleavage of substrates. These are shown in Figure 2. 

The catalytic domain is characterized by a consensus sequence “HEXXHXXGXXH”, which allows the linking of a zinc ion. The presence of a zinc ion facilitates the binding of H2O molecules, thus providing the hydrolytic reactions of peptides and substrates [14]. Except for MMP-7, -26, and -23, all MMP family members display an hemopexin domain known to play a role in substrate recognition, proteolytic activity, and inhibitor binding. The GPI-anchored MT4-MMP displays unique features as compared to other MT-MMP members [15]. First, MT4-MMP is distantly related in its amino acid sequence to the other members. The catalytic domain displays less than 40% sequence identity, while the sequence identity is more than 65% among the other MMP members [1]. Second, MT4-MMP is unable to process pro-MMP2 into its active form, in contrast with MT1-, MT2-, MT3-, and MT5-MMP [5,6,16]. The pro-MMP2-activating MT-MMPs contain eight amino acids located in the catalytic domain, the so-called “MT-loop”, which are lacking in MT4-MMP [17]. It has been reported that the pro-MMP2 activation is impaired when the MT-loop of MT1-MMP is deleted or inhibited by neutralizing antibodies [18]. These results are consistent with the capacity of the MT-Loop of MT1-MMP to interact with the fibronectin-like domain of pro-MMP2. Furthermore, a mutation in the MT-Loop of MT1-MMP impairs pro-MMP2 activation [19]. Thirdly, unlike other MMPs, MT4-MMP has a small repertoire of substrates among the ECM, with the exception of weak hydrolyzing capacities against fibrinogen, fibrin, and gelatin [5,6]. However, MT4-MMP is efficient in the cleavage of proTNF, ADAMTS4, α-2-macroglobulin, low density lipoprotein receptor-related protein, osteopontin, and cartilage oligomeric matrix protein, as detailed in Table 1 [2,5,6,8,20,21]. Interestingly, a soluble MT4-MMP form has been abundantly detected in the media of cells overexpressing active MT4-MMP, but not in the media of cells overexpressing its inactive form [22]. These data suggest that MT4-MMP is able to process itself by an active autocleavage. Finally, MT4-MMP is most potently inhibited by TIMP1 rather than TIMP2 or TIMP3, unlike the pro-MMP2-activating MT-MMPs that are efficiently inhibited by TIMP-2, -3, and -4, but not by TIMP-1 [6,7].

## 3. Biosynthesis and Trafficking

**Figure 3:**
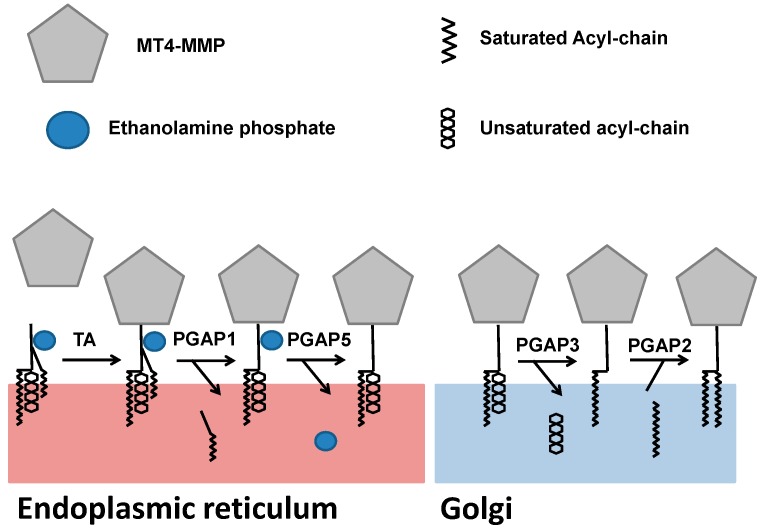
The biosynthesis of the glycosylphosphatidylinositol anchor on the MT4-MMP.

MMPs are synthetized as inactive zymogens. The inactive form is maintained by the interaction between the cysteine sulfhydryl group in the pro-domain and the zinc ion bound to the catalytic domain. The activation of MMPs requires a proteolytic cleavage of their pro-domain [26]. The MT4-MMP contains a furin consensus sequence (R–X–K/R–R) and can be activated by furin [7,27]. The precursor form (69 kDa) is found in the ER and in the Golgi compartment, while the processed form (58 kDa) is present at the membrane [12]. The biosynthesis of GPI proteins such as MT4-MMP follows a unique pathway that starts in the ER and finishes in the Golgi [28]. The mature GPI protein is finally transported to the cytoplasmic membrane by endoplasmic vesicles. In the lumen of the ER, the nascent protein is attached to a preformed GPI present in the inner membrane of the ER by a GPI transamidase. The preformed GPI is then modified following different steps to reach a mature form. The acyl chain is removed by PGAP1 (Post-GPI attachment to proteins 1) and the ethanolamine phosphate chain is released by PGAP5. After arrival in the Golgi, the unsaturated acid is replaced by a saturated fatty acid under the actions of PGAP3 and PGAP2, as illustrated in Figure 3 [28]. The fatty acid remodeling is essential for the incorporation of GPI-anchored proteins into lipid rafts, conferring to them the ability to interact with and degrade specific components of the raft environment. During this process, MT4-MMP is also N-glycosylated at the Asn318 site [12].

**Figure 4:**
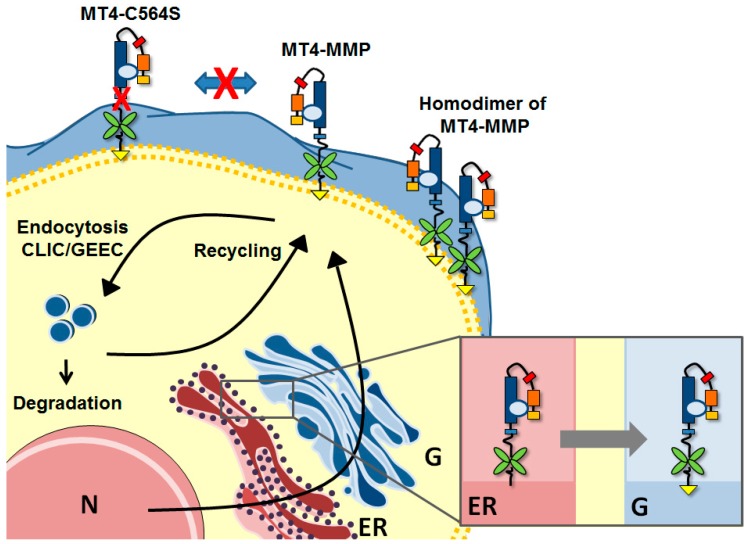
Biosynthesis and trafficking of MT4-MMP. MT4-MMP is associated to the glycosylphosphatidylinositol anchor in the endoplasmic reticulum (ER) and the Golgi (G) before being brought to the membrane. MT4-MMP can form homodimers, but the switch of cysteine with a serine prevents the dimerization. The protease is internalized by the clathrin-independent carriers/GPI-enriched early endosomal compartments pathway (CLIC/GEEC). In the cytoplasm, the protease is degraded or recycled at the membrane. N: Nucleus.

While MT4-MMP has been shown to colocalize with caveolin-1, a major structural protein associated with lipid rafts in mammalian cells, MT4-MMP internalization is independent of the caveolin-1 pathway [29]. Indeed, its internalization is not disturbed by filipin III, a caveolae pathway inhibitor. Similarly, treatment with chlorpromazine, an inhibitor of the clathrin pathway, does not block MT4-MMP internalization. Moreover, no colocalization of MT4-MMP and clathrin has been observed. MT4-MMP internalization involves a clathrin-independent carriers/GPI-enriched early endosomal compartments (CLIC/GEEC) pathway commonly used for GPI-anchored protein endocytosis, which is shown in Figure 4 [29]. The silencing of CDC42, Rac1, and RhoA, three regulators of the CLIC/GEEC endocytic pathway, disturbs MT4-MMP internalization. This implication of the CLIC/GEEC pathway in MT4-MMP internalization is up till today unique in the MMP family, further underlying the specific features of this membrane-associated enzyme. MT4-MMP present at the cell surface is internalized in early endosomes, and part of the enzyme is intracellularly autodegraded or recycled to the cell surface [29]. We and others have demonstrated the key role of the cysteine residue C564 in MT4-MMP dimerization, whereas the effect of its demonization on its activity is still unknown [29,30]. 

## 4. Physiological Expression and Functions of MT4-MMP

The spatiotemporal expression of *mt4-mmp* during murine embryonic development has been linked to its key role in angiogenesis, limb development, and brain formation [31]. The implication of MT4-MMP in the neuronal system is not limited only to the brain, but also involved in the migration of neural crest cells during embryogenesis of the zebrafish. Indeed, its inhibition by morpholino or by broad spectrum MMP inhibitors (Marimastat and ONO-4817) results in aberrant neural crest cell migration, with minimal changes in cell proliferation or apoptosis [32]. In transgenic mouse models expressing different types of retinal ganglion neurons with distinct directional migration, *mt4-mmp* transcripts have been identified in retinal ganglion cells with preferences for nasal motion, but not for ventral motion [33]. These data suggest a role of MT4-MMP in the orientation of the retinal ganglion neuron migration [34]. Overall, MT4-MMP-deficient mice grow normally and display a normal appearance, behaviour, life span, and fertility [21]. The unique observable effect in this model is a hypodipsia with a decreased daily urine output. While the kidney function is normal and MT4-MMP is expressed in the hypothalamus, which regulates thirst, it has been proposed that MT4-MMP may play a role in thirst regulation [35]. The expression of MT4-MMP is prominent in the brain, lungs, and uterus, and is transitional in the spleen, stomach, intestines, and reproductive organs [1,21]. MT4-MMP is also detected in human eosinophils, lymphocytes, monocytes, and macrophages, suggesting a role of this protease in inflammation [1,6,9,21,36,37]. Its expression in eosinophils is increased upon stimulation with TNF-α [37]. While the expression of MT4-MMP has been linked to different physiological processes, the mechanisms underlying these processes are still unknown. In contrast, more investigations were dedicated to understanding the role of this protease in pathologies, and several mechanisms were unravelled and will be described in this review and summarized in Table 2. The mechanism of action of MT4-MMP is not only related to its proteolytic activity, but also to its non-proteolytic function. We previously demonstrated that MT4-MMP is a key precursor and partner of Epidermal Growth Factor Receptor (EGFR), and enhances its activation leading to cancer cell proliferation in a non-proteolytic activity [38]. Future studies aiming at targeting these two functions will further define its relevance in cancer and diseases. 

## 5. Osteoarthritis

Articular cartilage function depends on ECM homeostasis that is dependent on a fine-tuned equilibrium between synthetic and degradative processes. Osteoarthritis (OA) is characterized by inflammation and a degradation of cartilage resulting from an increase of aggrecanase activity. Accordingly, MT4-MMP has been described with an aggrecanase activity, suggesting a role in cartilage homeostasis [2]. In OA, both *mt4-mmp* mRNA and the protein were found upregulated in this pathology [24,39]. *Mt4-mmp* transcripts are detected in human OA cartilage, but not in intact control cartilage [24]. Patwari et al. demonstrated that interleukin-1 (IL-1) considerably increases MT4-MMP expression in articular cartilage disk explants obtained from the femoropatellar groove of calves and in the conditioned medium [23]. Similar results were observed in C57BL6/Jax mice injected with IL-1 [24]. MT4-MMP is able to cleave the ADAMTS4 p68 isoform, which possesses relatively poor aggrecanase activity, to generate the ADAMTS4 p53 isoform, which is the highly active form [2]. In inflammatory conditions, the amount of total ADAMTS4 protein is not modulated. However, the ADAMTS4 p53 isoform is more abundant than the p68 isoform, reflecting an increaseof aggrecanase activity. The generation of the p53 isoform is directly linked to MT4-MMP expression. Indeed, MT4-MMP null mice are protected from IL-1-induced cartilage aggrecanolysis [24]. All these data imply the role of MT4-MMP in the regulation of aggrecan processing under inflammatory contexts, and open new therapeutic perspectives in osteoarthritis treatment.

## 6. Thoracic Aortic Aneurysms and Dissections

The role of MT4-MMP in the regulation of vessel stability in cancer and vascular diseases is well established [4,22,40]. More recently, its role in the thoracic aortic aneurysms and dissections (TAAD) has been reported in an elegant study by Martin-Alonso and colleagues, who performed screening of 58 patients with inherited predispositions to TAAD and identified a missense mutation R373H in the *mt4-mmp* gene that prevents the expression of the protease [8]. In a genetic mouse models of MT4-MMP loss of function, dilated aortas are observed, with dysfunctional vascular smooth muscle cells (VSMCs) and ECM changes [8,21]. Moreover, these mice are hypotensive and display an adventitial fibrosis as described in other mouse models of TAAD [41]. Furthermore, MT4-MMP expression is detected in periaortic progenitors during embryogenesis, suggesting a role of MT4-MMP in the early construction of the aortic wall and VSMC maturation. While no TAAD are observed spontaneously in MT4-MMP-deficient mice, its incidence is increased in MT4-MMP-null mice compared to the wild type mice when vessel wall stress is induced by angiotensin-II treatment. In another model of vascular injury triggered by ligation of the carotid artery, MT4-MMP-null mice display an alteration of vascular remodelling, characterized by a neointima more prominent in the carotid arteries and increased VSMC proliferation. Among the vascular substrates of MT4-MMP, osteopontin has been identified. This protein is expressed in the vessel wall, including in the embryonic aorta, and is associated with VSMC migration and differentiation, and more interestingly with aortic aneurysm [42,43,44,45]. Parallel to the increase of osteopontin fragments mediated by MT4-MMP cleavage in the aorta, an increase of c-Jun N-terminal kinase (JNK) phosphorylation is observed, highlighting a signalling pathway activated by MT4-MMP during VSMC maturation. Finally, the restoration of MT4-MMP expression by lentivirus partially rescues the vessel-wall phenotype, providing the way for new therapeutic strategies [8,25]. Atherosclerosis can be associated with TAAD [46,47]. Interestingly, the absence of MT4-MMP expression in mice is correlated with the recruitment of patrolling monocytes and lipid deposits in atherosclerotic plaques. Indeed, MT4-MMP cleaves αM integrin at the surface of crawling monocytes, inducing their detachment and the loss of their function in the regulation of atherosclerosis [9].

## 7. Cancer

### 7.1. Gastric Cancer

Wang et al. investigated the expression of *mt4-mmp* transcripts and proteins in 42 cases of gastric cancer and normal tissues, and 40 cases of atrophic gastritis [48]. Interestingly, no difference in MT4-MMP expression is observed between normal tissues and atrophic gastritis cases. However, its expression is higher in gastric cancer patients than in normal and atrophic gastritis tissues. This study highlighted an association of MT4-MMP expression with the depth of tumor invasion, lymph node metastasis and serosal involvement of gastric cancer patients [48]. In an experimental model of gastric cancer cell lines exposed to oxidative stress in vitro, the expression of MT4-MMP was increased among other MMPs and β-catenin, suggesting a potential role of this enzyme in the pathogenesis of gastric cancer, dependent on the continuous exposure of the mucosa to oxidative stress [49].

### 7.2. Colon Cancer

MT4-MMP is expressed in lipid rafts of highly metastatic colon cancer cell line HM-7, but not in the parental lower metastatic cell line LS174T, suggesting a role of MT4-MMP in the metastatic dissemination of colon cancer [11]. Inversely, caveolin-1 is not expressed in metastatic HM-7 cells and weakly expressed in the cytosolic fraction of parental LS174T cells. Interestingly, the restoration of caveolin-1 expression in metastatic HM-7 cells inhibits MT4-MMP expression in the lipid rafts, suppressing the metastatic phenotype of colon cancer cells. While the role of caveolin-1 in MT4-MMP trafficking has been excluded [29], the impact of caveolin-1 on MT4-MMP expression could be explained by other mechanisms, including the regulation of *mt4-mmp* transcription or translation, or the release of MT4-MMP from the membrane by proteases or phospholipases, which all can be regulated by caveolin-1 activity [11].

### 7.3. Head and Neck Cancer

Huang et al. described an unprecedented link between hypoxia and the regulation of MT4-MMP expression in head and neck cancer [3]. The experiments on hypopharyngeal squamous cell carcinoma (FADU) and tongue squamous cell carcinoma (SAS) showed an increase of *mt4-mmp* transcripts and proteins in hypoxic conditions or under constitutive expression of HIF-1. In reverse, HIF-1 silencing decreases MT4-MMP expression. Among the key regulators of hypoxia, SLUG has been identified as the major factor responsible for hypoxia and HIF-1α-induced MT4-MMP expression. Indeed, SLUG activates the transcription of *mt4-mmp* through direct interaction with the E-box located in the promoter of the *mt4-mmp* gene. Interestingly, MT4-MMP silencing reduces HIF-1α- or SLUG-induced invasiveness and lung dissemination of cancer cells. In a retrospective clinical study, MT4-MMP and HIF-1α colocalized in 20 out of 68 patients, and the colocalization of the both proteins was associated with poor overall survival [3]. 

### 7.4. Breast Cancer

Chabottaux et al. investigated the expression of MT4-MMP in 21 samples of healthy breast tissue and 63 breast adenocarcinomas [13]. Immunohistochemistry staining revealed a higher expression of MT4-MMP in breast adenocarcinomas, while healthy breast tissues presented a negative to moderate positivity [13]. Interestingly, the overexpression of MT4-MMP in MDA-MB-231 cells (triple-negative breast cancer cell line) promotes cell proliferation in the 3D matrix in vitro and in subcutaneous xenografts [13,38]. Moreover, MT4-MMP expression induces lung metastases by destabilizing blood vasculature, characterized by an enlargement of blood vessel lumens and pericyte detachment [4]. While no differences in the production of key angiogenic modulators (VEGF, PDGFR, FGF, and their receptors) were detected, expression of human thrombospondin-2 (TSP-2) is decreased in MT4-MMP xenografts. This result fits with the decrease of this anti-angiogenic factor that has been associated with impaired vascular integrity and permeability in mouse models [50]. Moreover, genetic deletion of TSP-2 in knock-out mice favors tumor growth, metastasis, and angiogenesis [51,52]. Interestingly, Salz et al. found that the transcription of different MMPs, including *mt4-mmp*, is regulated by hSETD1A, a methyltransferase overexpressed in metastatic human breast cancer cell lines and patients [53]. The silencing of hSETD1A decreases H3K4 methylation in promoters, decreasing MMP transcription and leading to decreased cell migration and invasion in vitro, and a reduction of lung metastases in mice [53]. Host et al. provided evidence for the requirement of proteolytic activity for MT4-MMP-mediated proangiogenic and prometastatic effects [22]. In sharp contrast, MT4-MMP has been reported to exert mitogenic effects on triple-negative breast cancer cells that are independent of its proteolytic activity. Indeed, MT4-MMP stimulates cell proliferation by interacting with EGFR and enhancing its activation in response to its ligands, Epidermal Growth Factor (EGF) and Tumor Growth Factor (TGF) [38]. Moreover, superimposition of EGFR and MT4-MMP has been observed in human triple-negative breast cancer (TNBC) patients [54]. Interestingly, MT4-MMP expression has been recently identified as a biomarker for TNBC patient responses to chemotherapy and to a combination of anti-EGFR drugs such as Erlotinib and Palbociclib, an inhibitor of Cyclin-dependent kinases 4 and 6, which are involved in the cell cycle [54,55]. These recent data highlight the clinical relevance of using the MT4-MMP/EGFR axis to select patients who could benefit from specific combinations of targeted therapies. 

## 8. Clinical Inhibitors of MMPs

Almost thirty years ago, MMPs were viewed as interesting targets for therapeutic compounds. Rationally, the TIMPs, endogenous inhibitors of MMPs, have been considered as potential treatments. Unfortunately, the development of clinical drugs has been aborted due to technical difficulties with the production and use of these proteins [56]. Also, studies on TIMPs have revealed dual functions in inhibiting or promoting cancer progression through several mechanisms involving intracellular signalling that are independent from their inhibitory activity of MMPs [57,58,59]. Other natural inhibitors have been taken into consideration, such as Neovastat, a molecule extracted from shark cartilage, which prevents angiogenesis and metastases. These anti-tumor effects are not only due to the inhibition of MMPs, but also the inhibition of VEGF [60]. Another natural compound is Genistein, a soy isoflavonoïd that blocks tumor growth and invasions by altering, among other things, the expression and the activity of MMPs and TIMPs [61,62]. Rapidly, synthetic inhibitors of MMPs have been produced and tested in different clinical trials [63]. However, those clinical trials using broad-spectrum MMP inhibitors were disappointing despite the promising preclinical studies. The clinical benefit was not convincing, and the secondary effects were intolerable [64]. The new generation of MMP inhibitors are designed to be more selective to decrease the secondary effects [65]. Monoclonal antibodies specifically directed against the catalytic domains of MT1-MMP or MMP-9 were promising in vitro and in vivo [66,67]. To date, only monoclonal anti-MMP-9 (GS-5745; Gilead Science) is being investigated in clinical trials. More recently, an antibody directed towards active MMP-13 has been produced and characterized [68]. Interestingly, MT4-MMP is emerging as a marker of interest to select patients who could benefit from specific combinations of existing treatments [55]. These findings hold new promise in the field of MMPs.

## 9. Discussion and Perspectives

The specific roles of MMPs and their inhibitors in cancer progression are widely recognized [69]. Although the family of human MMPs is composed of 25 enzymes, only five MMPs, including four soluble forms (MMPs-1, -2, -9, and -13) and a membrane form (MT1-MMP), were intensively studied. This interest in these MMPs is linked to their overexpression identified on the basis of the genomic and transcriptomic data collected on different types of human cancers [70]. Because of its membrane localization and its pericellular proteolytic action, MT1-MMP has been the subject of intensive studies that have elucidated its primordial role in the migration of cancer cells [71,72]. The search for scientific publications in the national center for biotechnology information (NCBI) database by combining the terms “MT1-MMP” and “cancer” reveals 340 articles from 1998 to the present. On the other hand, only 60 scientific articles are proposed for MT4-MMP in cancer. The low interest for this protease is due to the fact that the overexpression of MT4-MMP was not detected in transcriptomic studies. However, analyses by immunohistochemistry showed that the protein is very abundant in the tumor compartment of human breast cancers [13,38,54]. Several hypotheses can be envisaged, such as: (1) The existence of several transcripts resulting from alternative splicing that would not be detected in the transcriptomic analyses, (2) post-transcriptional regulation via miRNAs, (3) translation control, and/or (4) epigenetic mRNA changes [73]. Since 2006, special attention has been brought to the study of MT4-MMP, which has been identified in experimental models of mammary cancers as a driver of metastasis [4,13]. In addition, this protease has unique biochemical characteristics that distinguish it from other MMPs, suggesting different functions [15]. First, MT4-MMP is anchored to the membrane by a GPI anchor, a feature only shared with MT6-MMP, and second, unlike most MT-MMPs, MT4-MMP is inefficient in the activation of pro-MMP2 and hydrolyses very few ECM components [5,6]. Moreover, its sensitivity to TIMPs is different from that of other MMPs [6,7]. One of the plausible explanations for this specificity of MT4-MMP is the low-percentage sequence homology of its catalytic domain compared to other MMPs [1]. All these data suggest that MT4-MMP presents unique functions and different substrates, promoting different pathologies, particularly in tumor progression. All these data support the development of blocking molecules to counter the effects of MT4-MMP. The pathological functions of this protease depend mainly on its proteolytic activity. Interestingly, the catalytic domain of MT4-MMP possesses a distinct sequence to the other MMPs, allowing the development of specific inhibitors as function-blocking antibodies.

## Figures and Tables

**Table 1:** Overview of identified substrates of MT4-MMP.

Substrates	References
ECM substrates:	
Gelatin	Wang et al., 1999 [5]
Fibrin	English et al., 2000 [6]
Fibrinogen	English et al., 2000 [6]
Other substrates:	
proTNF	Wang et al., 1999 [5]
	English et al., 2000 [6]
COMP	English et al., 2000 [6]
α-2-macroglobulin	English et al., 2000 [6]
LRP1	Rozanov et al., 2004 [20]
ADAMTS4	Gao et al., 2004 [2]
	Patwari et al., 2005 [23]
	Clements et al., 2011 [24]
MT4-MMP	Host et al., 2012 [22]
Osteopontin	Martin-Alonso et al., 2015 [8]
	Papke et al., 2015 [25]
Thrombospondin 4	Martin-Alonso et al., 2015 [8]
αM integrin	Clemente et al., 2018 [9]

**Table 2:** Proteolytic and non-proteolytic functions of MT4-MMP in different pathologies.

Diseases	Proteolytic Functions	Non-Proteolytic Functions	Unknown
Breast cancer	Angiogenesis ^1,2^, Metastatic dissemination ^1,2^	Cell proliferation ^3^	
Colon cancer			Metastatic dissemination ^4^
Head and neck cancer			Metastatic dissemination ^5^, Hypoxia ^5^
Osteoarthritis	Processing of ADAMTS4 p68 isoform ^6,7,8^		
Thoracic aortic aneurysms and dissections	Processing of osteopontin ^9,10^		
Atherosclerosis	Processing of αMintegrin ^11^		

^1^ Chabottaux et al., 2009 [4], ^2^ Host et al., 2012 [22], ^3^ Paye et al., 2014 [38], ^4^ Nimri et al., 2013 [11], ^5^ Huang et al., 2009 [3], ^6^ Gao et al., 2004 [2], ^7^ Patwari et al., 2005 [23], ^8^ Clements et al., 2011 [24], ^9^ Martin-Alonso et al., 2015 [8], ^10^ Papke et al., 2015 [25], ^11^ Clemente et al., 2018 [9].
